# Beyond fluorescence intensity: a Minimum acquisition and reporting standard and emerging technologies for colorectal indocyanine green fluorescence angiography studies

**DOI:** 10.3389/fsurg.2026.1864848

**Published:** 2026-07-02

**Authors:** Parvat Kuwar Chhetri, Anmol Bhan, Kristen K. Rumer, David W. Larson

**Affiliations:** Department of Surgery, Division of Colorectal Surgery, Mayo Clinic, Rochester, MN, United States

**Keywords:** anastomotic leak (AL), anastomotic perfusion, artificial intelligence (AI), colorectal surgery, hyperspectral imaging (HSI), indocyanine green (ICG) fluorescence angiography, laser speckle contrast imaging (LSCI), standardization

## Abstract

**Background:**

Intraoperative indocyanine green fluorescence angiography (ICG-FA) is increasingly utilized to assess bowel perfusion during colorectal anastomosis. Although recent multicenter randomized trials have yielded mixed findings, pooled randomized evidence and 2025 SAGES guidance indicate an overall benefit, particularly in left-sided and rectal surgery. However, acquisition protocols, interpretation criteria, and reported endpoints remain heterogeneous.

**Main message:**

ICG-FA reflects the dynamics of an intravascular dye signal and is affected by injection technique, hemodynamic status, camera geometry, display mode, and platform-specific processing. Visual interpretation shows substantial interobserver variability. Thus, single fluorescence-intensity snapshots are unstable for comparison. In contrast, time-intensity kinetics and spatial heterogeneity measures are more reproducible and physiologically meaningful. Among reported kinetic metrics, the time ratio (TR = T1/2Max/Tmax) is promising. However, current evidence is preliminary and needs multicenter validation.

**Proposal:**

We propose a practical minimum acquisition and reporting standard for colorectal ICG-FA studies as an expert-opinion framework for future consensus development. This includes consistent reporting of ICG administration, physiological status at assessment, imaging conditions, camera geometry, analysis methodology, measured outcomes, decision impact, and software/version details. We also describe other relevant approaches, such as laser speckle contrast imaging (LSCI), hyperspectral imaging (HSI), and artificial intelligence (AI)-assisted interpretation.

**Conclusions:**

The key question is no longer only whether ICG-FA should be used, but how its signal should be acquired, quantified, interpreted, and reported. Better standardization should improve comparability, support multicenter validation, and clarify where perfusion imaging adds the greatest clinical value.

## Introduction

Anastomotic leak (AL) is one of the most consequential complications in colorectal surgery. It is associated with increased morbidity, mortality, reoperation, permanent stoma, longer hospital stays, higher costs, and delayed adjuvant chemotherapy (1, 2). AL rates vary by procedure type, anastomosis level, and case-mix. Compromised bowel perfusion at the anastomosis is among the few potentially modifiable intraoperative risk factors (3).

Fluorescence-guided surgery has expanded rapidly in colorectal practice. Indocyanine green (ICG) fluorescence angiography (ICG-FA) is most often used to evaluate anastomotic perfusion. ICG is an FDA-approved, water-soluble tricarbocyanine fluorophore. After intravenous injection, it quickly binds to plasma proteins and emits near-infrared (NIR) fluorescence. This signal is detectable by NIR-capable cameras, enabling real-time, intracorporeal visualization of tissue perfusion during minimally invasive or open procedures (4, 5). Despite widespread adoption and strong face validity, the clinical impact of routine ICG-FA remains difficult to define in individual trials. However, pooled RCT evidence now supports an overall benefit, particularly in left-sided and rectal surgery (6).

Four major randomized controlled trials (RCTs) have recently reported results. The Japanese Essential trial enrolled 850 patients for minimally invasive sphincter-preserving rectal cancer surgery. It showed a significant reduction in overall AL with ICG-FA, but the absolute difference (4.2 percentage points) did not meet the pre-specified 6-point threshold (7). The Dutch AVOID trial (*n* = 982), which evaluated all colorectal resection types, found no significant reduction in grade B/C (clinically relevant) AL at 90 days with ICG-FA-guided assessment vs. conventional assessment. However, a subgroup analysis of left-sided resections suggested potential benefit (8). The Finnish ICG-COLORAL trial (*n* = 1,136), which excluded low anterior resections, also found no significant overall reduction in AL with routine ICG-FA (9). Most recently, the European IntAct trial enrolled patients with rectal cancer across 28 centers in eight countries. It reported only a trend toward reduced AL that fell short of statistical significance

 (10). The most recent RCT-only meta-analysis included nine trials and 4,754 patients. It found that ICG-FA reduced overall and clinically significant anastomotic leaks, with the strongest evidence in the left-sided, rectal, and low anterior resection subgroups. Right-sided benefit remained uncertain (6). The central question is no longer whether ICG-FA can be beneficial, but rather how its signal should be acquired, interpreted, and standardized to reproduce benefit across studies and clinical settings.

We contend that ICG-FA is most effective as an intraoperative safety measure rather than a standalone strategy for preventing anastomotic leaks. AL is a multifactorial complication, with perfusion representing only one aspect of anastomotic healing, alongside factors such as anastomotic tension, technical integrity, host nutritional and metabolic status, and microbiome influences (11, 12). Therefore, a negative trial of ICG-FA should not be interpreted as evidence that perfusion is unimportant; rather, it may reflect the inability of optimizing a single modifiable factor to fully mitigate risk from others. Furthermore, null results from studies employing simplified fluorescence variables should not be construed as evidence against the value of perfusion imaging. It is essential to distinguish between the limitations of intraoperative tests in predicting multifactorial complications and the validity and reproducibility of the fluorescence variables used to assess perfusion. Variability in acquisition protocols and reporting across centers can obscure clinically meaningful associations, even when impaired perfusion contributes to risk. In this context, we review the limitations of conventional ICG-FA metrics, summarize emerging quantitative and alternative perfusion technologies, and propose a pragmatic minimum acquisition and reporting standard to support more reproducible and objective decision-making.

## Perfusion biology versus perfusion measurement: A critical distinction

Perfusion measurement does not fully capture perfusion biology. ICG-FA visualizes the transit of an intravascular dye bolus rather than directly measuring tissue oxygenation or microvascular function. A bright fluorescence signal, therefore, indicates dye delivery and filling, but it may not fully represent the oxygenation state of the bowel segment that must heal after anastomosis. This distinction is clinically important because a visually reassuring signal can still provide an incomplete picture of perfusion status when interpretation relies solely on intensity (13).

Against this biological background, many colorectal ICG-FA studies still emphasize maximum intensity and simple timing measures such as time-to-visibility and Tmax. Here, time-to-visibility denotes first visible fluorescence; Tmax, time to maximum fluorescence intensity; T1/2MAX, time to half-maximum intensity; inflow slope, rate of fluorescence rise; AUC, the area under the time-intensity curve; and TR, the ratio (T1/2MAX)/(Tmax). These measures are influenced by acquisition conditions, patient-specific factors, and anatomic context. Ahn et al. showed that Fmax, inflow slope, and T1/2MAX varied significantly with camera distance and imaging mode. Gomez-Rosado et al. reported higher Fmax values in patients with arterial hypertension and in more distal anastomoses (14). On multivariable analysis, TR was the only quantitative parameter not significantly associated with the conditional acquisition factors studied. This suggests it may be more robust across variable intraoperative conditions, though this remains preliminary and needs prospective multicenter validation (15, 16).

Although normalization strategies and ratio-based parameters, such as TR, can partially mitigate certain sources of technical variation, routine documentation of acquisition conditions and relevant physiological covariates remains essential for meaningful cross-study interpretation. A critical additional concern is that broadly transferable reference ranges for “vital” tissue perfusion remain incompletely established across devices, software pipelines, and imaging protocols, making it difficult to generalize thresholds for “adequate” perfusion (17).

Several colorectal studies report links between anastomotic complications and curve-derived kinetic features. These include inflow slope, T1/2MAX, and TR (15, 18). Son et al. found that TR strongly discriminated between leak and non-leak on receiver-operating characteristic analysis (AUC 0.929). TR > 0.6 was an independent factor in logistic regression (18). They found that T1/2MAX and Tmax correlated with tissue oxygen saturation measured by hyperspectral imaging. Fmax showed no correlation with StO₂ in their regression model (13).

In summary, the future utility of ICG-FA in colorectal surgery depends more on capturing the dynamic and spatial variability of fluorescence than on single-intensity measures. This approach is more closely aligned with physiological assessment and informed clinical decision-making.

## Interobserver variability and the imperative for quantitative kinetics

A growing body of literature documents that the visual interpretation of colorectal ICG-FA is variable even among experienced colorectal surgeons. Hardy et al. found limited concordance in binary judgments of perfusion sufficiency from ICG-FA videos, with interpretation influenced by experience and operative context, supporting the need for objective quantification (19). In robot-assisted low anterior resection, Larsen et al. reported wide interobserver variation for visually assessed timing parameters, whereas *post hoc* pixel-based quantification identified a lower fluorescence time-curve slope in patients who later developed anastomotic leak (20). Similarly, Faber et al. showed that ROI-based quantification can differentiate distinct fluorescence time–intensity curve patterns, whereas the surgeon’s subjective interpretation demonstrated only poor-to-moderate interobserver agreement (21).

Taken together, these studies argue for moving beyond subjective assessment or single-value intensity summaries toward standardized, curve-based kinetics (e.g., inflow slope, T1/2MAX, AUC, and outflow/washout features) and more systematic reporting of spatial heterogeneity along the bowel edge (15).

A significant unresolved challenge, however, is the lack of agreement between different quantification software implementations (algorithms). A comparative analysis by Nijssen et al. found a mean relative difference of more than 58% in normalized maximum slope between two independently developed algorithms applied to the same ICG-FA recordings, with increasing proportional bias at higher values (22).

This issue extends beyond a technical inconvenience. If identical videos produce substantially different quantitative outputs across software packages, thresholds established in one study cannot be reliably applied to other centers or platforms. This “algorithm gap” represents a significant barrier to multicenter validation and the development of clinically applicable decision thresholds.

Therefore, generalizable decision support will require both consensus on quantification methodology and inter-software harmonization (15, 22). As a practical step, the field should prioritize shared reference datasets and, ideally, open-source reference implementations against which commercial or institutional algorithms can be benchmarked.

## Emerging technologies: beyond conventional ICG-FA

Given the limitations of conventional ICG-FA, including its reliance on contrast injection, restricted repeatability within a single operative episode, sensitivity to acquisition conditions, and interobserver variability, several complementary and alternative perfusion imaging technologies have emerged. These modalities may inform future strategies for enhancing anastomotic safety (see Table 1).

**Table 1:** Summary of perfusion imaging modalities discussed and their practical trade-offs.

Modality	Input/equipment	Primary output	Key strengths	Key limitations/current maturity
ICG-FA (visual)	ICG bolus; NIR-capable platform	Qualitative fluorescence pattern; surgeon judgment	Widely available; minimal workflow disruption; enables immediate revision of transection line	Clinically available/evidence-supported adjunct in selected settings. Subjective; sensitive to acquisition conditions and physiology; repeat assessment requires reinjection; thresholds not standardized
ICG-FA (quantitative)	ICG bolus; recorded video; software analysis	Time–intensity kinetics (e.g., slope, T1/2MAX, AUC) and spatial heterogeneity metrics	More objective than visual; supports audit and data pooling; enables algorithm development	Early translational. Software/platform variability; requires standardized acquisition and validation; real-time deployment remains limited
LSCI	Dedicated LSCI system; no dye required	Superficial blood-flow map (speckle-derived)	Continuous and immediately repeatable; provides complementary flow information; avoids dye injection	Early clinical/translational. Motion sensitivity; shallow penetration; limited human colorectal outcome validation
HSI	Dedicated HSI system; no dye required	Tissue oxygenation and hemoglobin-related maps (StO₂, HbO₂/Hb)	Provides contrast-free oxygenation information; may help define perfusion border; complements dye- and flow-based measures	Early clinical/investigational adjunct. Slower acquisition; motion artifacts; workflow integration varies; outcome thresholds still evolving
AI-assisted interpretation	Imaging input, usually ICG-FA video; trained software/model	Automated interpretation/scoring; in some systems, segmentation and decision support; standardized documentation	May reduce interobserver variability; may standardize expert-style interpretation; enables structured datasets	Development-stage/investigational. Requires prospective multicenter validation; generalizability across devices must be shown; dataset bias, governance, regulatory, and medicolegal issues remain

## Laser speckle contrast imaging

Laser speckle contrast imaging (LSCI) provides continuous, dye-free, real-time assessment of superficial tissue blood flow by analyzing dynamic speckle patterns generated when coherent laser light interacts with moving red blood cells. Unlike ICG-FA, it can be repeated immediately and allows continuous monitoring, but current reviews emphasize that clinical adoption is still early and that standardized acquisition and interpretation protocols have not yet been established (23, 24). In colorectal surgery, Skinner et al. showed that advanced visualization with LSCI and ICG-FA altered intraoperative decision-making in 17.5% of minimally invasive left-sided colorectal resections, while the LSCI-defined ischemic line did not differ significantly from the ICG-FA-based line of demarcation (25). In a prospective pilot study of anterior rectal resection, Yu et al. found no overall perfusion difference between matched high-tie (division and ligation of inferior mesenteric artery at its aortic origin) and low-tie (division and ligation below the origin of the left colic artery) groups, but LSCI identified a small high-risk subgroup within the high-tie group that led surgeons to revise the transection line in 16.7% of cases (26). Overall, LSCI is promising, but the human colorectal evidence base is still small, and larger studies with standardized protocols are needed before its role in anastomotic decision-making can be defined (24).

## Hyperspectral imaging

Hyperspectral imaging (HSI) is another contrast-free optical technique that records tissue reflectance across visible and near-infrared wavelengths and generates spatial maps of tissue oxygenation and hemoglobin-related parameters (27). By providing an objective map of bowel oxygenation, HSI may help define the perfusion border and guide transection-line selection during colorectal resection (28, 29).

In a 115-patient colorectal series, Jansen-Winkeln et al. reported that the clinically chosen transection line matched the HSI-defined border in only 55.2% of cases, highlighting the limitations of visual assessment alone (29).

Barberio et al. further showed that HSI could directly influence intraoperative decision-making: in 26 of 52 patients, the hyperspectral and clinical transection lines differed, and when the discrepancy exceeded 5 mm, resection was performed at the HSI-defined line (28).

A practical limitation of many HSI systems is slower acquisition, which requires a relatively motionless field. In minimally invasive surgery, bowel movement from respiration and surgical manipulation may introduce motion artifacts and distort the perfusion map. Although newer HSI systems now offer shorter acquisition times than earlier open systems, current measurements still take several seconds and remain susceptible to motion artifact, which limits workflow integration (30, 31). HSI is therefore a promising complementary modality for objective oxygenation mapping, but its role in minimally invasive colorectal surgery still requires further technical refinement and clinical validation (30, 31).

## Artificial intelligence and automated perfusion interpretation

AI-assisted analysis is a promising extension of quantitative ICG-FA because it can automate interpretation and reduce reliance on subjective visual judgment. Mc Entee et al. described AUGUR-AI, a real-time system that combines colon auto-segmentation with expert-annotated ICG-FA interpretation. In that study, the interpretation model achieved intersection-over-union (IoU) scores of 0.72 in training and 0.62 in testing; the auto-segmentation model achieved an IoU of 0.86; and complete live deployment was demonstrated across 15 surgeries (32). These overlap metrics support technical feasibility and proof-of-concept deployment, but they are not sufficient to justify stand-alone clinical decision-making. He et al. developed an XGBoost model using biophysical features extracted from fluorescence kinetics and achieved an R² of 97.2% for clinician-scored perfusion quality (33), while Park et al. used self-organizing map learning to classify ICG curves into 25 patterns and reported more consistent prediction of anastomotic complication risk than conventional parameters (34).

Collectively, these studies indicate that AI may facilitate standardized interpretation and documentation. However, current models remain vulnerable to dataset bias, platform dependency, limited explainability, regulatory review and medicolegal uncertainty. Broader prospective validation across institutions and imaging platforms is necessary before AI-based perfusion support can be widely implemented.

## A pragmatic Minimum acquisition and reporting standard

To clarify when and in which patients ICG-FA changes outcomes, we propose that future trials and prospective registries adopt a practical minimum acquisition and reporting standard. McEntee et al. found marked heterogeneity in acquisition methods, parameter selection, and software across colorectal ICG-FA quantification studies, with only four real-time studies and one in which quantification informed intraoperative transection (15). Mirza et al. likewise identified the lack of standardized, objective interpretation criteria and validated quantitative thresholds as major barriers to reproducible clinical translation (35).

Building on these observations, we propose a concise minimum dataset (Table 2) that standardizes ICG administration, imaging conditions, and data capture to the extent feasible, while also recording relevant physiologic variables at the time of assessment. At minimum, we recommend reporting time-intensity kinetics and, where feasible, spatial heterogeneity measures rather than isolated intensity snapshots alone. Where ICG-FA is used alongside other anastomotic assessments, studies should also report those tests and specify whether any operative revision was driven by ICG-FA, adjunct testing or surgeon judgment. Ahn et al. showed that acquisition conditions, such as camera distance and imaging mode, materially affect the quantitative parameters measured, reinforcing the need for standardized protocols (16). Accordingly, for studies reporting quantitative endpoints, protocol-controlled or measured/calibrated camera-to-target distance is preferable to visual estimation, because commonly used parameters vary with camera position. Where direct measurement is impractical, studies should prespecify a reproducible calibration method. As practical implementation options, studies may use an in-field reference of known dimensions or, in robotic systems, explore platform-derived spatial measurements where available. Ahn et al. derived distance using a ruler-based method and recommended a constant acquisition distance of 4–5 cm, whereas Faber et al. used a fixed 30 cm, 90° setup in a standardized colorectal video protocol (16, 21).

**Table 2:** A pragmatic research framework intended to improve comparability across trials, registries, and prospective cohort studies of colorectal ICG-FA. It should be viewed as a starting framework for future consensus development and prospective validation.

Domain	Proposed items to standardize/report
ICG administration	Dose per injection (mg; mg/kg if weight-based), concentration, IV site (peripheral vs central), flush volume, number/timing of injections, and predefined t = 0 for time-based analysis.
Physiology at assessment	Mean arterial pressure, heart rate, oxygen saturation, and vasopressor/inotrope use (and dose, if applicable) at the time of injection/assessment; hemoglobin/hematocrit if available.
Imaging system/settings	Platform and camera model; intracorporeal vs extracorporeal acquisition; NIR mode; exposure/gain (locked vs auto); excitation/illumination source if known; software version; post-processing filters if used.
Camera geometry	Working distance, angle, and zoom/lens setting at assessment; specify whether the camera was fixed or freehand and whether distance was protocol-controlled, directly measured, or derived from a predefined calibration method (e.g., an in-field reference of known dimensions or platform-derived spatial measurement).
Assessment site/analysis method	ROI-based vs pixel-wise/full-field analysis; site relative to the intended transection line; mesenteric vs antimesenteric side if relevant; ROI size, shape, number, and any reference ROI.
Data capture	Save ICG-FA video, ideally with timestamps, starting before or at injection and continuing through complete inflow and peak. If delayed-peak, outflow, or washout endpoints are planned, record long enough to capture those phases. Save white-light/overlay video if available.
Quantitative endpoints (if used)	Prespecified primary endpoint(s); exact formula/derivation for each metric; raw vs normalized curves; at least one clearly defined timing/inflow metric; exact definition of heterogeneity or washout metrics if used; software name/version and preprocessing steps.
Decision impact	Whether ICG-FA changed the surgical plan, when, and how (for example transection revision, additional resection, anastomotic revision, or proximal diversion); additional bowel length revised/resected if measured;
Adjunct modalities/AI (if used)	Whether LSCI, HSI, or AI-assisted interpretation was used; platform/model and version; real-time vs *post hoc* output; whether it contributed to the final intraoperative decision.
Other anastomotic assessment (if used)	Whether additional anastomotic testing was performed (e.g., Air leak test, flexible sigmoidoscopy, Doppler). If final decision was driven by surgeon judgment, ICG-FA(visual only, quantitative analysis or both), other anastomotic testing or a combination.

We also recommend explicit documentation of the quantification software or algorithm used, its version, and any preprocessing or calibration steps, given the demonstrated inter-software variability in quantitative output. In a human clinical dataset, Nijssen et al. reported a mean relative difference of +58.2% in normalized maximum slope between two independently developed software implementations applied to the same standardized recordings, with increasing proportional bias at higher values (22). Routine saving of timestamped ICG-FA video can also support *post hoc* quantitative analysis and external validation; most published colorectal quantification studies to date have relied on recorded video (15) and ongoing multicenter AI-validation work similarly depends on pseudonymized video archives linked to clinical data (32).

This standard does not imply that ICG-FA alone can prevent anastomotic leaks; rather, it is intended to ensure that studies evaluate ICG-FA using comparable variables that better reflect the perfusion signal being measured.

## Discussion

Conflicting findings from individual multicenter RCTs should not be read as a final negative verdict on perfusion imaging in colorectal surgery. Instead, they highlight the difficulty of linking a single intraoperative assessment, acquired with variable methods and under variable physiologic conditions, to a downstream complication with multiple causes (15, 35). The minimum acquisition and reporting standard proposed here is intended to make future studies more comparable, facilitate pooled analysis and external validation, and support prospective validation of clinically useful thresholds and models; this need is reinforced by the randomized evidence itself, in which ICG-FA dose, timing, and imaging platform varied substantially across trials (6).

The strongest contemporary pooled evidence now comes from the most recent RCT-only systematic review and meta-analysis, which included nine trials and 4,754 patients and found that ICG-FA reduced overall anastomotic leak (RR 0.66, 95% CI 0.56–0.78), clinically significant leak (RR 0.73, 95% CI 0.60–0.89), and 30-day leak (RR 0.50, 95% CI 0.39–0.64). Benefit was strongest in left-sided, rectal, and low anterior resection subgroups, whereas right-sided benefit remained uncertain (6). These findings are consistent with the 2025 SAGES guideline, which issued a strong recommendation, with moderate certainty of evidence, for intraoperative ICG use in left-sided colorectal anastomosis (1, 36). The pooled RCT data also support the view that the benefit of ICG-FA is mediated mainly through intraoperative decision change rather than any direct biologic effect of the dye: a change in transection level occurred in 7.9% of ICG-FA cases. Conversely, the lack of benefit from delayed leaks reinforces the notion that perfusion is only one component of the multifactorial biology of anastomotic failure (6).

Given that the evidence for overall, left-sided, rectal, and low anterior resection outcomes is now mature, future research should shift from broad efficacy trials toward implementation studies, protocol standardization, and better subgroup definition (6). First, acquisition protocols and reporting standards must be practical for routine use (15, 37). Second, quantitative endpoints need prospective validation, with transparent definitions and demonstration of generalizability across imaging platforms, software, and patient populations (15, 16, 22). Third, prospective multicenter repositories of intraoperative perfusion imaging records linked to clinical covariates and outcomes would support *post hoc* analysis, external validation, and algorithm development (15, 32). Future studies should prespecify subgroup analyses where benefit may differ, including left- vs. right-sided resections, colorectal vs. colocolonic anastomoses, elective vs. emergency surgery, and patients with prior radiation treatment. Adjunct technologies such as LSCI, HSI, and AI-assisted interpretation should be studied in harmonized protocols rather than as isolated feasibility tools (15, 37). Real-world adoption will also depend on device cost, software licensing, training requirements, and institutional access to quantitative, HSI, and AI platforms.

Standardized datasets also create a practical foundation for automated interpretation. AUGUR-AI has already shown that real-time AI-assisted interpretation can be deployed in the operating room using an edge device and zero-touch workflow (32). Other groups have also shown that machine-learning models can predict clinician-scored perfusion quality from fluorescence kinetics and classify ICG curve patterns more consistently than conventional single-parameter approaches (33, 34). If prospectively validated across centers and platforms, such tools could reduce interobserver variability, support training, and improve documentation, while preserving the pragmatic role of ICG-FA as an intraoperative safety check rather than a stand-alone solution for anastomotic leaks (38). This standardization agenda should also extend to complementary perfusion modalities, as comparative colorectal data suggest that HSI and ICG-FA can identify similar transection margins, while meta-analytic evidence supports an association between intraoperative bowel perfusion assessment and lower anastomotic leak rates (39, 40).

In summary, the primary question is no longer whether ICG-FA should be used, but rather how its signal should be acquired, quantified, interpreted, and reported. Improved standardization will not eliminate anastomotic leaks independently, but it will enable more rigorous evaluation of perfusion imaging, facilitate reliable comparison of results, and help delineate its greatest clinical value.

### Limitations

This article presents a narrative perspective and an expert opinion rather than a systematic review. The proposed minimum acquisition and reporting standard has not undergone Delphi consensus, stakeholder review, or society endorsement and should therefore be interpreted as a practical starting framework for future consensus development and prospective validation rather than a definitive guideline.

## Data Availability

The original contributions presented in the study are included in the article/Supplementary Material, further inquiries can be directed to the corresponding author.
